# Gold nanoparticles approach to detect chondroitin sulphate and hyaluronic acid urothelial coating

**DOI:** 10.1038/s41598-017-09872-0

**Published:** 2017-09-04

**Authors:** Gabriella Guelfi, Valentina Stefanetti, Danilo Zampini, Oommen P. Oommen, Gabriele Brecchia, Cecilia Dall’Aglio, Rolando Arcelli, Giovanni Cochetti, Andrea Boni, Ettore Mearini

**Affiliations:** 1 Department of Veterinary Medicine, University of Perugia, Perugia, PG Italy; 2 Faculty of Biomedical Sciences and Engineering & BioMediTech Institute, Bioengineering and Nanomedicine Lab, Tampere University of Technology, 33520 Tampere, Finland; 3 Department of Surgical and Biomedical Sciences, Section of Urological, Andrological and Minimally invasive techniques, University of Perugia, Piazza Lucio Severi 1, 06132 Perugia, PG Italy

## Abstract

This study investigated the location of hyaluronic acid (HA)- and chondroitin sulphate (CS)-coated gold nanoparticles in rabbit bladder and evaluated gene expression of CD44, RHAMM and ICAM-1 receptors involved in HA and CS transport into the cell. Gold nanoparticles were synthesised by reduction of gold salts with HA or CS to form HA-AuNPs and CS-AuNPs. Bladder samples were incubated with CS-AuNPs and HA-AuNPs or without glycosaminoglycans. Transmission electron microscopy, optic microscopy and scanning electron microscopy were used to determine the location of the synthesised AuNPs. Real-time PCR was used to analyse expression of urothelial cell receptors CD44, RHAMM, ICAM-1, after *ex vivo* administration of CS-AuNPs and HA-AuNPs. We showed that HA-AuNPs and CS-AuNPs were located in the cytoplasm and tight junctions of urothelial umbrella cells; this appearance was absent in untreated bladders. There were no significant differences in gene expression levels for CD44, RHAMM and ICAM-1 receptors in treated versus control bladder tissues. In conclusion, we clearly showed the presence of exogenous GAGs in the bladder surface and the tight junctions between umbrella cells, which is important in the regeneration pathway of the urothelium. The GAGs-AuNPs offer a promising approach to understanding the biophysical properties and imaging of urothelial tissue.

## Introduction

Bladder epithelium, also known as urothelium or transitional epithelium, is a highly specialised tissue that plays a key role in the defence of the bladder wall against various toxins. The urothelium is coated in a thick layer of glycosaminoglycans (GAGs) that acts as a non-specific anti-adherence factor and protects against infection^1^. This GAG layer is embedded in a network of proteins, forming the so-called glycocalyx^2^. Although the function of this layer is not yet fully understood, it has been suggested that it forms a physical barrier with hydro-repellent properties that protects epithelial cells against the irritative effects of urine components^3^. In fact, the high density of GAGs allows water molecules to form a surface impenetrable to many low molecular weight solutes^4^. There is strong evidence that an absence of GAGs is linked to loss of normal urothelial function. This loss of function results in “storage symptoms” such as frequency, which is an innate protective mechanism designed to minimise contact between urine and the damaged bladder wall^5^.

Hyaluronic acid (HA) and chondroitin sulphate (CS) are two naturally occurring GAGs abundantly present on bladder urothelium^6^. Many of the effects of HA are mediated through cell surface receptors, three of which have been molecularly characterised, namely cluster of differentiation 44 (CD44), hyaluronan-mediated motility receptor (HMMR), also known as RHAMM, and intercellular adhesion molecule-1 (ICAM-1)^7^. Although CD44 is the principal HA receptor, it also shows an affinity for CS^8^. The CD44 affinity of these polymers has resulted in the development of several nanoparticles for cancer targeting^9^ and bulk hydrogel scaffolds for tissue engineering^10^.

Administration of exogenous GAGs is widely accepted as therapy in various bladder disorders^11, 12^. Intravesical instillation of HA and CS shows promise as a treatment for some bladder diseases, by promoting regeneration of GAG in the bladder urothelium^13, 14^. However, data are lacking regarding the localisation of these molecules within the urothelium and the extent of their integration to cells or tissues.

In order to improve the detectability of these molecules in tissue samples, we have engineered HA-and CS-coated gold nanoparticles (AuNPs)^15^, which have unique surface plasma resonance and optical properties^16^. In this study, we investigated the location of HA and CS after *ex vivo* incubation of rabbit bladder tissue with HA-AuNPs and CS-AuNPs to determine whether the benefits of HA and CS replacement therapy might be due to coating of the urothelium with GAGs. In addition, we evaluated relative gene expression of receptors involved in HA and CS transport into the cell (CD44, RHAMM and ICAM-1) in order to understand whether incubation with GAG nanoparticles modifies receptor expression.

## Materials and Methods

### Ethics Statement

The procedures involving the animals and their care were conducted in conformity with the national and international laws and policies. Animal protocols were reviewed and approved by the Animal Committee of the University of Perugia, Italy, and followed the guidelines for the care and use of animals at our institution (Comitato Universitario di Bioetica, permit number 129/2016-PR). All rabbits used for the study were provided by Centro Servizi per la ricerca pre-clinica (Perugia, Italy). All the animals were housed in each cage and fed with water and food *ad libitum*.

### Synthesis of CS and HA coated AuNPs

CS 38 kDa and HA 1500 kDa were provided by IBSA, Italy. This particular molecular weight HA was chosen to best mimic *in vivo* conditions. Gold chloride and NaBH_4_ were purchased from Sigma-Aldrich. HA-AuNPs and CS-AuNPs were synthesised using the NaBH_4_ reduction method, as described by Tengdelius *et al*.^17^ with slight modifications. Briefly, gold chloride was dissolved in 10 mL of deionised water and refluxed for two hours then cooled to 60 °C. CS 30 mg (1 equivalent with respect to disaccharide units) was dissolved separately in 10 mL of deionised water. For the synthesis of HA-coated AuNPs, 24 mg (1 equivalent with respect to disaccharide units) of HA was used. The HA or CS solution was added to the gold solution (kept at 60 °C) and NaBH_4_ (4.56 mg, 0.12 mmol, 2 equivalents) was added. The reaction was left to progress for 8 hours at 60 °C and the formation of NP was observed immediately as the colour changed to deep violet. Thereafter, the CS-AuNP and HA-AuNP solutions were loaded into a dialysis bag (MWCO 3500), dialysed against deionised water (4 × 2 L) for 48 h and lyophilised to obtain 30 mg of CS-AuNPs or 25 mg of HA-AuNPs. The compounds were stored at room temperature and before use were re-suspended in water at a final concentration of 1 mg/mL.

### Synthesis of BSA-AuNP

BSA-AuNP used as a control in the study was obtained by incubating commercially available citrate coated gold nanoparticles (40 nm, from EY Laboratories, Inc.) with BSA following the reported protocol^18^. The concentration of BSA used for coating was same as the two GAGs used to synthesize HA and CS AuNPs.

### Nanoparticle characterisation

CS-AuNPs and HA-AuNPs were monitored using spectrophotometric measurements (Evolution 201 UV VIS; Thermo Fisher). Transmission electron microscopy (TEM; Philips EM 208 equipped with a digital camera) measurements were made from samples prepared by placing drops of the gold NP dispersions on carbon-coated TEM copper grids (Società Italiana Chimici-Roma); mixtures were allowed to dry for 1 minute then excess solution was removed using blotting paper. Dynamic light scattering (DLS) measurements were carried out using Malvern’s Zetasizer Nano ZS instrument.

### Animals and tissue preparation

Eighteen 8-week-old healthy New Zealand female White rabbits (≈1500 ± 200 g) were used in the study. Animals were sedated with 0.2 mg/kg medetomidine (Medetor; VirbacSrl) and 10 mg/kg intramuscular ketamine (Inoketam; VirbacSrl) and anaesthetised with 5% isofluorane. Rabbits were euthanised by injection of 200 mL ketamine/xylazine until breathing stopped. Bladders were harvested (weighing 5–6 g) and washed extensively with sterile phosphate-buffered saline (PBS) supplemented with penicillin 200 U/mL, streptomycin 200 mg/mL, and amphotericin-B 12.5 mg/mL (Sigma-Aldrich; St. Louis, USA) to remove debris. Tissue was then placed in 5 mL of Dulbecco’s Modified Eagle Medium (DMEM, Gibco; Gaithersburg, USA), 10% foetal bovine serum (Gibco, Gaithersburg, USA), penicillin 10,000 U/mL, streptomycin 10 µg/mL and amphotericin-B 10 µg/mL.

### GAGs treatment

Twelve rabbit bladder sections (weighing 0.7 to 0.9 g) were incubated with 500 µL of CS-AuNPs (1 mg/mL) and 500 µL of HA-AuNPs (1 mg/mL) at 37 °C for 60 minutes; the other six samples received the same treatment but were incubated with colloidal gold solution adsorbed with bovine serum albumin instead of GAGs (BSA-AuNPs; control). The 60-minute incubation time was adopted as during this time, no significant necrosis or cell degeneration as observed.

### Sample preparation for microscopy

Bladder samples for optical microscopy were fixed by immersion in 2.5% glutaraldehyde in 0.1 M PBS at room temperature for 24 hours. Then the tissue samples were dehydrated through a graded series of ethanol, cleared in xylene and embedded in paraffin. Sections were processed for a better identification of the bladder epithelium using haematoxylin-eosin (HE) staining (Sigma-Aldrich, St. Louis, Mo, USA).

For scanning electron microscopy (SEM; Philips XL 30 resolution: 2.0 nm at 30 kV and 10 mbar water vapour), small bladder samples were fixed in 2.5% glutaraldehyde in distilled water for 2 hours at room temperature. Samples were washed in distilled water for 20 minutes, and dehydrated through ascending grades ethanol, according to the critical point drier method. Dried specimens were mounted on stubs with silver paste and sputtered with gold (200–250 nm) by cathode atomisation under vacuum.

For TEM, small samples of bladder tissue were placed in 2.5% glutaraldehyde added to 0.1 M PBS, pH 7.3, for 3 hours at room temperature. After rinsing twice in PBS, the specimens were post-fixed in 1% buffered osmium tetroxide at pH 7.2 for 3 hours at 4 °C. Samples were then dehydrated, pre-infiltrated and embedded in Epon 812. Semi-thin sections (1 µm) were obtained and stained with methylene blue and Azur blue II^19^ and examined under a light microscope to identify the best specimens. Ultrathin sections were mounted on 200-mesh copper grids, stained with uranyl acetate and lead citrate^20^.

### Evaluation of receptor RNA expression

RNA was extracted from 5 µm paraffin-embedded bladder sections adjacent to that observed using optical microscopy. Extraction was performed with a commercial kit (RecoverAll Total Nucleic Acid Isolation Kit; Ambion, Austin, USA) following the manufacturer’s instructions. Total RNA was quantified with a fluorometer (Qubit 2.0, Invitrogen, Carlsbad, USA). About 20 ng of total RNA were reverse transcribed (RT) in 20 µL of iSCRIPTcDNA (BioRad; Hercules, USA) using random hexamers. No-RT controls were included to check for genomic DNA contamination.

Quantitative real-time polymerase chain reaction (qRT-PCR) analysis was carried out with 5 μL of a ten-fold diluted cDNA in a final volume of 25 μL using 10 μL of SsoFast™ EvaGreen1 Supermix (BioRad; Hercules, USA). Primers for CD44, RHAMM and ICAM-1 were commercially purchased (PrimePCRSyberGreen Assay, Bio-Rad; Hercules, CA, USA).

All PCR reactions (Bio-Rad iCycler Real-Time PCR) had an initial incubation at 95 °C for 15 minutes, followed by 45 cycles at 95 °C for 15 seconds and 60 °C for 1 minute, during which fluorescence data were collected. Each sample was run in triplicate and the results were averaged. Sample amplification fidelity was verified by agarose gel electrophoresis. 18 S rRNA was used as an endogenous control to normalise variations in the amount of starting RNA samples as previously reported^21^. For each PCR run, no template controls and no-RT controls were included in order to ascertain the absence of gDNA.

The 2^−ΔΔCt^ method was used to calculate the relative expression of the target genes as follows^22^:

$${2}^{-}(({{\rm{C}}}_{{\rm{t}}}{\rm{target}}-{{\rm{C}}}_{{\rm{t}}}{\rm{18S}})-({{\rm{C}}}_{{\rm{t}}}{\rm{control}}-{{\rm{C}}}_{{\rm{t}}}{\rm{18S}}))$$

### Statistical analysis

Results are presented as mean ± standard error (SE). Statistical comparisons between groups were performed using unpaired t-test. Significance was set at p ≤ 0.05. All analyses were performed in GraphPad Prism version 6.00.

## Results

### GAGs-AuNPs evaluation

**Figure 1:**
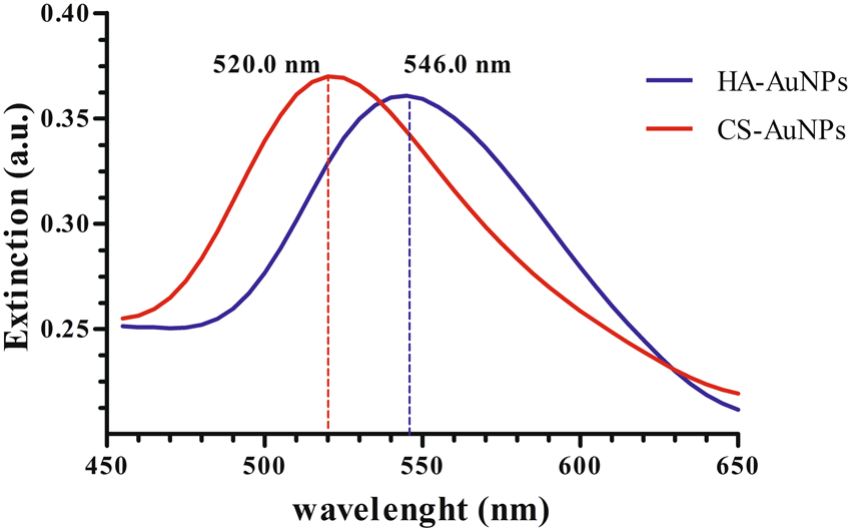
Gold colloids absorption spectrum with a peak at 546 nm for HA-AuNP (blu) and at 520 for CS-AuNP (red), showing how the reaction proceeds. This measurement establishes the optimum conditions for the preparation of gold colloids.

GAGs-AuNPs displayed a UV-Vis analysis at λ _max_ 546 nm (HA) and 520 (CS) single surface plasma resonance, suggesting formation of spherical morphology (Fig. 1).

**Figure 2:**
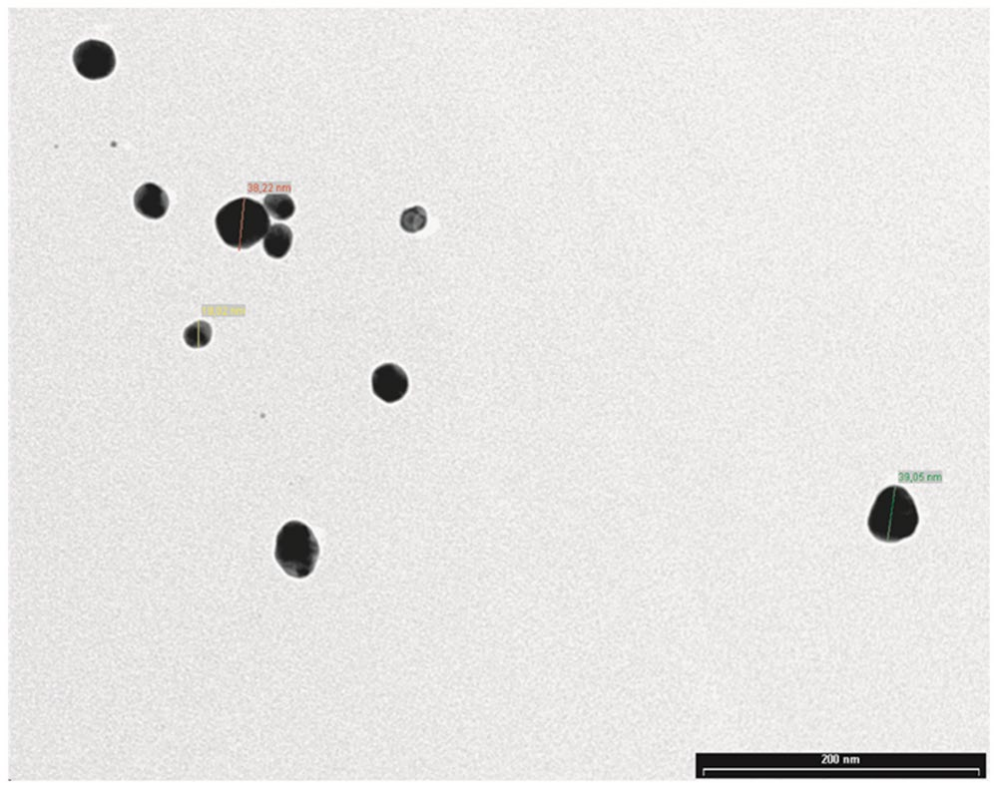
Transition electron microscopy (TEM) image showed both GAGs-AuNPs (19 to 40 nm), re-dispersed in water and deposited in the form of films onto carbon-coated TEM grids.

DLS analysis indicated that for HA-AuNPs 89% nanoparticles had a size of 98 ± 18 nm, while a zeta potential (Zp) = −36.5 ± 5 mV, and for CS-AuNPs 99.4% nanoparticles had a size of 58 ± 12, with a Zp = −23.5 ± 4 mV. On the contrary, TEM analysis indicated a 40 ± 1.5 nm and 19 ± 0.6 nm radius, for HA-AuNPs and CS-AuNPs respectively, as Fig. 2 shows.

The higher hydrodynamic size of the nanoparticles as determined by DLS are attributed to the hydration of GAG coat, which is not discernible in the TEM measurements.

### Microscopy evaluations

**Figure 3:**
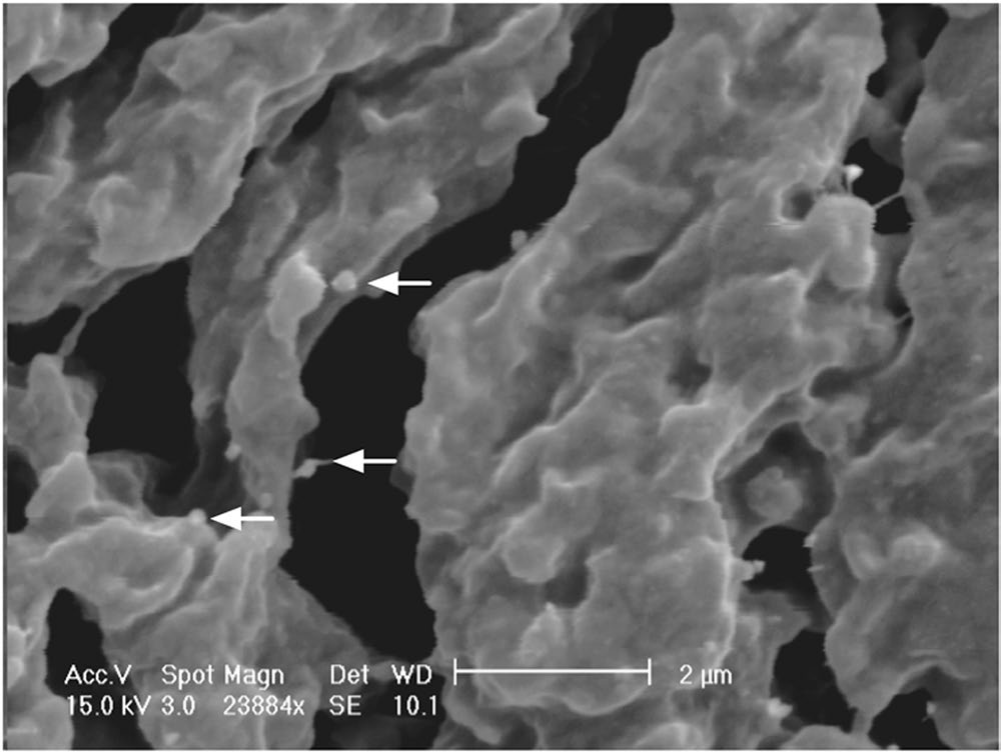
Scanning electron microscopy (SEM) image of treated bladder epithelium showing GAGs-AuNPs (arrow) linked to the plasma membranes of epithelial cells

**Figure 4:**
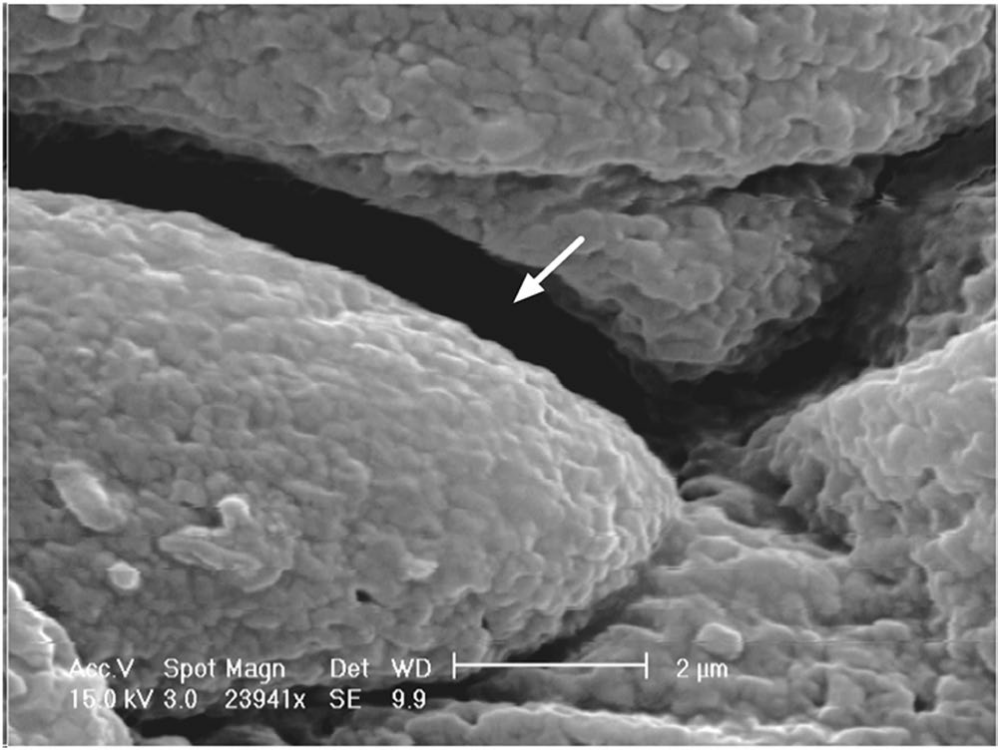
Scanning electron microscopy (SEM) image of untreated (control) bladder epithelium showing an absence of any visible aggregates.

After *ex vivo* incubation, SEM observations showed the vast majority of epithelial cells with a scalloped luminal face being covered by some ridges of GAGs-AuNPs (Fig. 3). This appearance was absent in untreated bladders (Fig. 4).

**Figure 5:**
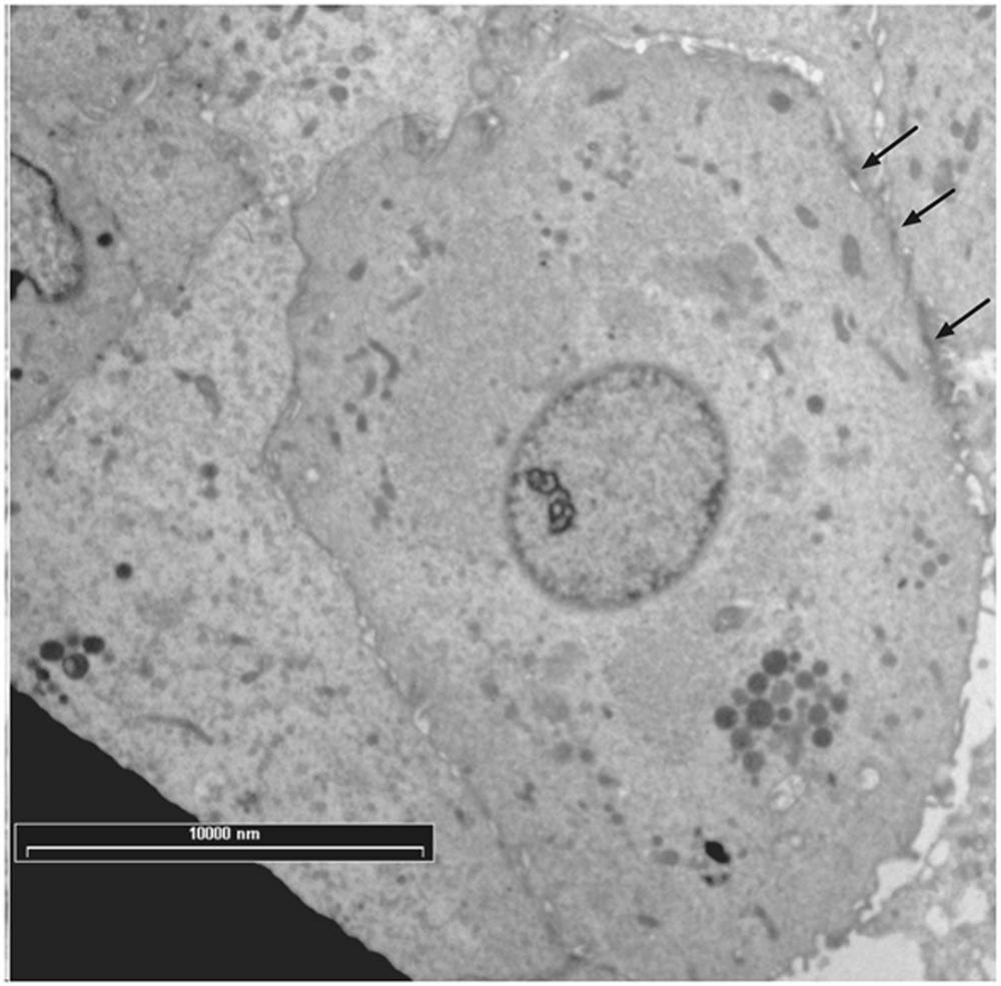
Transition electron microscopy (TEM) image showing GAGs-AuNPs linked to the plasma membrane of epithelial cells (arrows).

**Figure 6:**
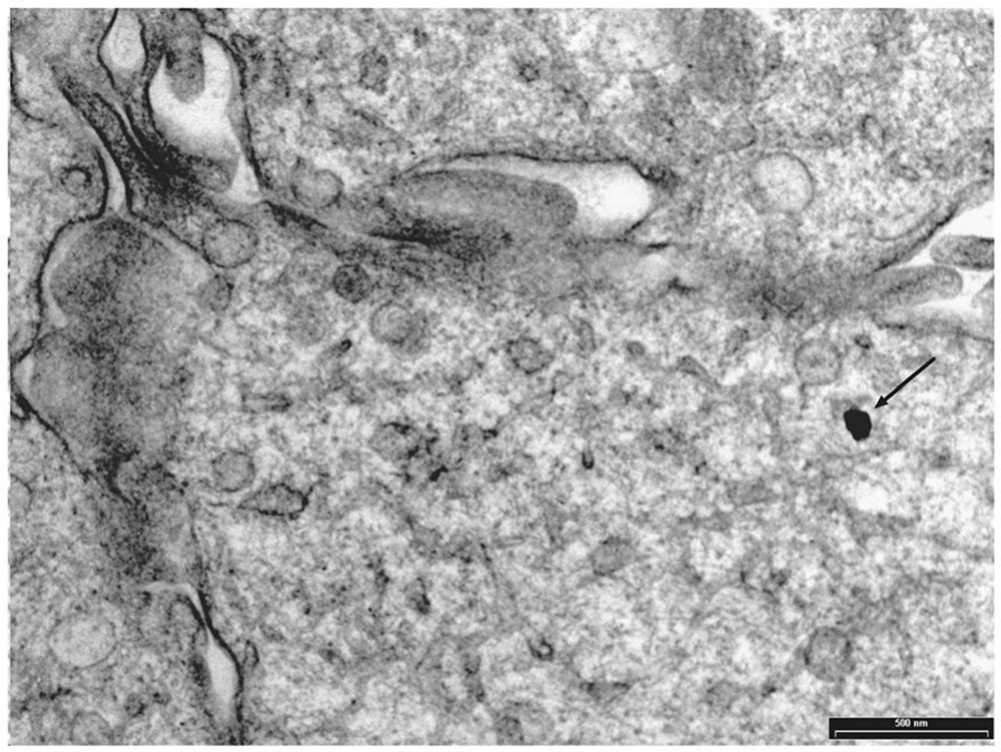
Transition electron microscopy (TEM) image showing GAGs-AuNPs linked to the plasma membrane of epithelial cells with some particles localised in the cytoplasm (arrows).

**Figure 7:**
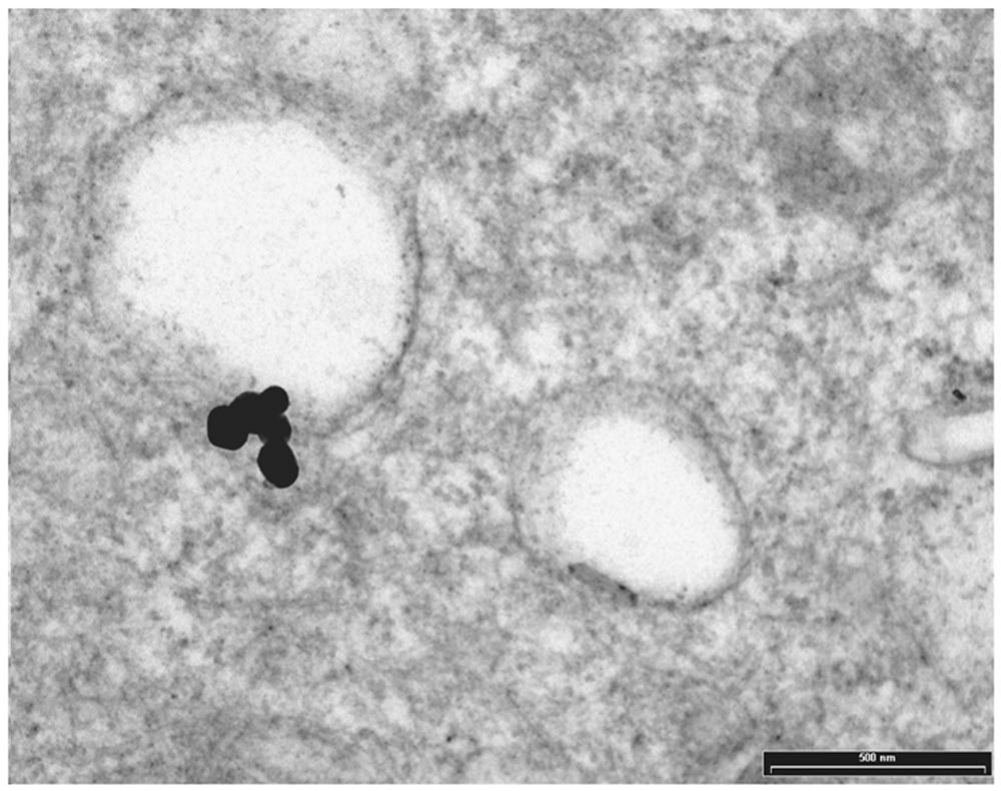
Transition electron microscopy (TEM) image showing GAGs-AuNPs linked to the plasma membrane of epithelial cells with some particles localised in the cytoplasm nearest to cellular membranous structures.

TEM analysis showed some particles of GAGs-AuNPs behind plasma membranes of apex line cells of the bladder epithelium (Fig. 5). In particular, some GAGs-AuNPs were evident in the cytoplasm (Figs 6 and 7), suggesting that nanoparticles can enter inside the cells, overcoming the plasma membrane barrier, and localise on the membranes of cytoplasmic organelles.

### PCR assessment

**Figure 8:**
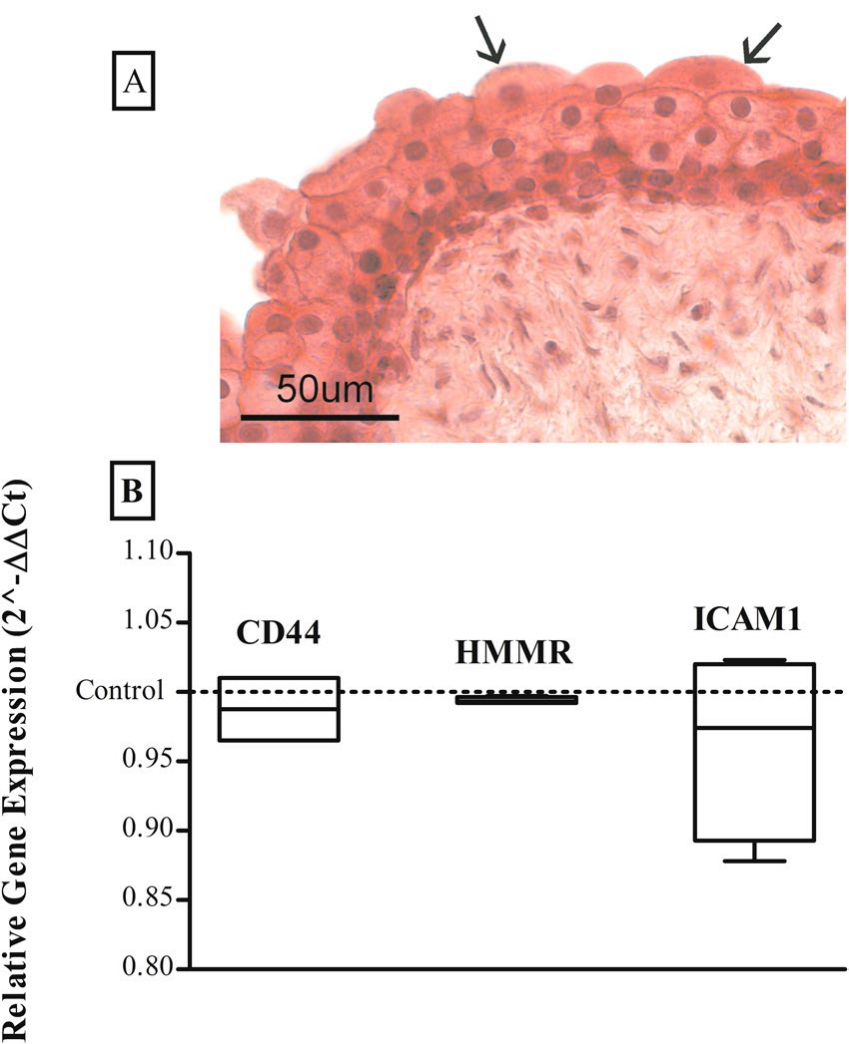
Light microphotograph (**A**) with haematoxylin and eosin (HE) stain showing histologic evaluation of rabbit bladder (Top to base: umbrella, intermediate and basal cells; arrows show the typical “dome-shaped” apical cells). Normalized expression value 2^−ΔΔCt^ (mean ± standard error) (**B**) of CD44, RHAMM and ICAM-1 in bladder tissues incubated with HA-AuNP and CS-AuNP (left box for each type of receptor) and control bladder tissues incubated with BSA-AuNPs solution (dashed line).

Single peaks in the melting curves confirmed the uniqueness of PCR products. There were no statistically significant differences in receptor gene expression in bladders incubated with HA-AuNP and CS-AuNP compared with control. The ICAM-1gene showed the highest mRNA expression level in treated group (1.83 ± 1.23) vs control (1.82 ± 0.56) while CD44 and HMMR gene expression in treated samples (0.74 ± 0.28 and 0.15 ± 0.05) were lower than those in control samples (0.56 ± 0.10 and 0.06 ± 0.02) (Fig. 8).

## Discussion

This study showed the presence of exogenous GAGs in bladder umbrella cells after *ex vivo* incubation of samples with gold nanoparticles containing CS and HA. No change in GAG receptor expression levels was detected in treated bladders.

The rabbit bladder is a good model for investigating the physiological, histological, and biochemical properties of the functioning human bladder^23^. Similarities include similar receptor distribution in the urinary tract and comparable pharmacological responses to a wide variety of agents^24^. The use of rabbit bladders offers several advantages, including availability of samples and transport of bladder tissue, and fewer ethical issues problems compared with the use of bladders from other species (e.g. dogs, pigs or humans).

We investigated the location of HA- and CS-coated gold nanoparticles in rabbit bladder tissue in order to demonstrate that the mechanism of action of GAGs replacement therapy is presumed to be due to a real coating of the bladder surface to replace physiological HA and CS lost as a result of disease. AuNPs are emerging as a very useful tool due to their unique electronic, optical, and catalytic properties, with simple synthesis, good stability and chemical inertness compared with other nanocarriers^25^. GAGs molecules with the sulphated polyanionic structure stabilise AuNPs in aqueous solution, thus improving biocompatibility of the metal^15^.

A GAG layer covers the umbrella cells and contributes to urothelial barrier function, also reducing the adhesion of many urinary bacterial pathogens. Furthermore, several events originating from intravesical GAG injury may result in chronic damage to the bladder epithelium and prevent healing due to direct contact of abnormal urinary constituents with the subepithelial layer. Prompt restoration of the GAG layer to prevent the inflammatory cycle is a rationale underlying treatment of chronic cystitis and painful bladder syndrome/interstitial cystitis^26^. “GAG replacement therapy” is effective in many patients, as shown in several models of urothelial damage, showing strong surface binding^11^. Another treatment option, based on endocavital GAG replenishment therapy has been shown to improve urinary irritative symptoms secondary to pelvic radiation therapy (RT)^27^, and to have potential as prophylactic therapy in this setting^28^. RT stimulates a neuroinflammatory cascade with activation of mast cell and release of histamine that could be inhibited by HA, while CS promotes regeneration of the AG layer^27^.

This study clearly showed the presence of exogenous GAGs in tight junctions and in the cytoplasm of umbrella cells. This is particularly important if we consider the regeneration pathway of the urothelium. In fact, within a few days of urothelial damage, tight junctions begin to develop between intermediate cells, increasing the water-tightness of the new layer. In this sense, the early or prophylactic administration of exogenous GAGs could play an important role in the rapid restoration of the urothelial impermeability^29^. In particular, the tight junction plays a leading role in the establishment of the transepithelial resistances, thus blocking ion flux within the epithelium. We also showed that HA and CS were able to penetrate into umbrella cells, which prompted us to evaluate intracellular signal transduction pathways using real-time PCR. Most of the effects of HA are mediated through CD44, RHAMM, and ICAM-1. Binding of the HA ligand to its receptors can direct cell trafficking during physiological and pathological events^30^. Our aim was also to demonstrate that the entry of HA and CS into umbrella cells is not associated with an increase in expression of CD44, RHAMM, and ICAM-1, and this was indeed the case. Southgate and colleagues^31^ reported CD44 expression in the basal layer of normal human urothelium that progressed from the basal to the superficial layer, where expression was lost. Similar CD44 expression profiling has been portrayed in rabbit bladder^32^. Further studies are warranted to facilitate understanding of the role of CD44, RHAMM, and ICAM-1 in the transitional epithelium.

A limitation of this research is that the microscopy analysis of GAG-AuNPs has only a qualitative value. As a result, we can only confirm the presence (not the quality) of GAGs-AuNPs on the urothelial mucosa. In addition, our aim was to investigate receptors on the surface of the urothelial cells and therefore we do not have information on the mechanism of uptake of GAG-AuNPs.

## Conclusions

This study clearly shows not only the presence of exogenous GAGs in the bladder surface but also in the tight junctions between umbrella cells, which is important in the regeneration pathway of the urothelium. Based on our findings, the entry of GAGs into urothelial cells does not seem to alter their receptor gene expression. The GAGs-AuNPs offer a promising approach to understanding the biophysical properties and imaging of urothelial tissue. In future, we will validate these results in an *in vivo* rabbit model and evaluate the administration of GAGs as a polymer and as AuNPs in an experimental model of cystitis, in order to study GAG-related molecular processes.

## References

[1] Damiano R, Cicione A (2011). The role of sodium hyaluronate and sodium chondroitin sulphate in the management of bladder disease. Ther Adv Urol.

[2] Wilson CB (1996). Extracellular matrix and integrin composition of the normal bladder wall. World J Urol.

[3] Poggi MM, Johnstone PA, Conner RJ (2000). Glycosaminoglycan content of human bladders. a method of analysis using cold-cup biopsies. Urol Oncol.

[4] Hurst RE, Zebrowski R (1994). Identification of proteoglycans present at high density on bovine and human bladder luminal surface. J Urol.

[5] Parsons CL (2007). The role of the urinary epithelium in the pathogenesis of interstitial cystitis/prostatitis/urethritis. Urology.

[6] Porru D (2012). Impact of intravesical hyaluronic acid and chondroitin sulfate on bladder pain syndrome/interstitial cystitis. Int Urogynecol J.

[7] Entwistle J, Hall CL, Turley EA (1996). HA receptors: regulators of signalling to the cytoskeleton. J Cell Biochem.

[8] Rudzki Z, Jothy S (1997). CD44 and the adhesion of neoplastic cells. Mol Pathol.

[9] Oommen OP (2016). Multifunctional hyaluronic acid and chondroitin sulfate nanoparticles: impact of glycosaminoglycan presentation on receptor mediated cellular uptake and immune activation. ACS Appl Mater Interfaces.

[10] Oommen OP (2013). Smart design of stable extracellular matrix mimetic hydrogel: synthesis, characterization, and *in vitro* and *in vivo* evaluation for tissue engineering. Adv Funct Mater.

[11] Lazzeri M (2016). Managing chronic bladder diseases with the administration of exogenous glycosaminoglycans: an update on the evidence. Ther Adv Urol.

[12] Fall M (2010). EAU guidelines on chronic pelvic pain. Eur Urol.

[13] De Vita D, Giordano S (2012). Effectiveness of intravesical hyaluronic acid/chondroitin sulfate in recurrent bacterial cystitis: a randomized study. Int Urogynecol J.

[14] Ciani O (2016). Intravesical administration of combined hyaluronic acid (HA) and chondroitin sulfate (CS) for the treatment of female recurrent urinary tract infections: a European multicentre nested case–control study. BMJ Open.

[15] Li W (2011). Facile synthesis of chondroitin sulfate-stabilized gold nanoparticles. Mater Chem Phys.

[16] Mandal S, Phadtare S, Sastry M (2005). Interfacing biology with nanoparticles. Curr Appl Phys.

[17] Tengdelius M (2015). Synthesis and anticancer properties of fucoidan-mimetic glycopolymer coated gold nanoparticles. Chem Commun Camb Engl.

[18] Brewer SH (2005). Probing BSA binding to citrate-coated gold nanoparticles and surfaces. Langmuir.

[19] Leeson CR, Leeson TS (1970). Staining methods for sections of epon-embedded tissues for light microscopy. Can J Zool.

[20] Venable JH, Coggeshall R (1965). A simplified lead citrate stain for use in electron microscopy. J Cell Biol.

[21] Peng XX (2012). Selection of suitable reference genes for normalization of quantitative real-time PCR in cartilage tissue injury and repair in rabbits. Int J Mol Sci.

[22] Livak KJ, Schmittgen TD (2001). Analysis of relative gene expression data using real-time quantitative PCR and the 2(-Delta Delta C(T)) Method. Methods.

[23] Balasteghin KT (2003). Experimental model of bladder instability in rabbits. Int Braz J Urol.

[24] Levin RM (1994). Rabbit as a model of urinary bladder function. Neurourol Urodyn.

[25] Muddineti OS, Ghosh B, Biswas S (2015). Current trends in using polymer coated gold nanoparticles for cancer therapy. Int J Pharm.

[26] Han XM (2015). The effects of intravesical therapy with hyaluronic acid for painful bladder syndrome: Preliminary Chinese experience and systematic review. Taiwan J Obstet Gynecol.

[27] Gacci M (2016). Bladder instillation therapy with hyaluronic acid and chondroitin sulfate improves symptoms of post radiation cystitis: prospective pilot study. Clin Genitourin Cancer.

[28] Hazewinkel MH (2011). Prophylactic vesical instillations with 0.2% chondroitin sulfate may reduce symptoms of acute radiation cystitis in patients undergoing radiotherapy for gynecological malignancies. Int Urogynecol J.

[29] Khandelwal P, Abraham SN, Apodaca G (2009). Cell biology and physiology of the uroepithelium. Am J Physiol Renal Physiol.

[30] Entwistle J, Hall CL, Turley EA (1996). HA receptors: regulators of signalling to the cytoskeleton. J Cell Biochem.

[31] Southgate J (1995). Patterns of splice variant CD44 expression by normal human urothelium *in situ* and *in vitro* and by bladder-carcinoma cell lines. Int J Cancer.

[32] Arafat HA, Wein AJ, Chacko S (2002). Osteopontin gene expression and immunolocalization in the rabbit urinary tract. J Urol.

